# Computed Tomographic Features of Bezoars and Other Gastrointestinal Foreign Bodies in Dogs and Cats: A Comparative Analysis

**DOI:** 10.3390/ani15091260

**Published:** 2025-04-29

**Authors:** Jongwon Koo, Kidong Eom, Jaehwan Kim, Jeongyun Jeong, Hongji Yoon, Minsu Lee, Jinsoo Park, Jongmun Cho

**Affiliations:** 1 Department of Veterinary Medical Imaging, College of Veterinary Medicine, Konkuk University, Seoul 05029, Republic of Korea; gueyvege@konkuk.ac.kr (J.K.);; 2 Daegu Animal Medical Center, Daegu 42185, Republic of Korea; 3 SD Animal Medical Center, Seoul 04580, Republic of Korea; 4 Haeden Animal Medical Center, Yangju-si 11492, Republic of Korea

**Keywords:** bezoar, gastrointestinal foreign bodies, intestinal obstruction, veterinary diagnostics

## Abstract

This study presents a comparative analysis of the computed tomographic (CT), radiographic, and ultrasonographic (US) characteristics of gastrointestinal foreign bodies, including bezoars, in dogs and cats, and evaluates their association with complications and clinical outcomes. A total of 33 cases (26 dogs, 7 cats) with surgically or endoscopically confirmed foreign bodies were reviewed, classified as bezoars (*n* = 15) or distinct foreign bodies (*n* = 18). CT features such as attenuation values, transition zones, and proximal-to-distal small intestinal diameter ratios were compared. Bezoars typically appeared as intraluminal masses with mottled gas patterns and indistinct boundaries (33.3% vs. 94.4%, *p* < 0.001) and were associated with longer clinical signs (median 14 vs. 5.5 days, *p* = 0.013), more frequent transition zones (92.3% vs. 41.7%, *p* = 0.011), and a greater diameter ratio (2.9 vs. 1.25, *p* = 0.012) across the transition zone. Radiographic and US evaluations were available in six bezoar cases; only one radiograph (17%) detected the bezoar, while US showed acoustic shadowing in four cases (67%). Six patients (18%) experienced adverse outcomes, with bowel wall ruptures significantly associated with poor prognosis (*p* < 0.001). These findings highlight the superior diagnostic performance of CT, particularly for bezoars, and emphasize the importance of identifying transition zones and bowel diameter ratios in assessing gastrointestinal foreign bodies and their associated risks. Early CT evaluation may thus facilitate timely intervention and improve clinical outcomes.

## Simple Summary

Gastrointestinal foreign bodies are common emergencies in dogs and cats, requiring rapid diagnosis and intervention. This study analyzed computed tomography (CT), radiographic, and ultrasonographic (US) findings in 33 patients (26 dogs, 7 cats) with surgically or endoscopically confirmed gastrointestinal foreign bodies. Two main types of foreign bodies were identified: bezoars (clumps of indigestible material) and discrete objects. Bezoars were more challenging to detect on CT (*p* < 0.001) as they closely resembled normal intestinal contents, delaying diagnosis. Additionally, radiographs identified bezoars in only one case (1/6, 17%), while US showed acoustic shadowing in 4/6 cases (83%). Animals with foreign bodies in the duodenum had higher complication rates, and bowel wall rupture was significantly associated with adverse outcome (*p* < 0.001). This study highlights the diagnostic characteristics of gastrointestinal foreign bodies across CT, radiography, and US, aiding clinicians in making more informed treatment decisions. By improving diagnostic accuracy, this research can contribute to quicker intervention, reduced suffering, and better patient outcomes in veterinary emergency medicine.

## 1. Introduction

Computed tomography (CT) has emerged as a powerful diagnostic tool in veterinary medicine, providing superior anatomical detail and multiplanar capabilities that address many limitations of conventional imaging modalities [1]. In gastrointestinal obstruction cases, CT offers critical information about the location, nature, and extent of foreign bodies and enables the assessment of potential complications [2,3]. Despite these advantages, interpreting CT findings in certain types of gastrointestinal foreign bodies remains challenging, particularly when the obstructive material lacks distinct boundaries or presents complex internal structures, as seen with bezoars [4].

Among gastrointestinal foreign bodies, bezoars are identified on CT by their mottled gas pattern, ovoid or round shape, and heterogeneous internal structure [4]. These masses of indigestible materials can be classified into several types based on their composition, including trichobezoars, phytobezoars, and pharmaco-bezoars [5,6,7,8]. While bezoars have been more extensively documented in human medicine, their clinical relevance in veterinary patients remains less well characterized. Behavioral tendencies such as pica—the ingestion of non-nutritive substances—are known to occur in dogs and cats [9], potentially contributing to a broader spectrum of foreign body types encountered in veterinary practice. Therefore, this study hypothesizes that bezoars of varying origins may be more diverse and prevalent in animals than previously recognized.

Gastrointestinal foreign bodies are common surgical emergencies in small animal medicine, presenting with varied clinical signs depending on the location, degree, and duration of the obstruction [10,11]. When complete obstruction occurs, it results in severe clinical symptoms and rapid deterioration of the patient’s condition. Partial obstruction, while less acute, also leads to significant complications, including chronic signs of maldigestion and malabsorption [11]. Furthermore, these foreign bodies can induce life-threatening complications. Obstruction-induced luminal distention impairs blood flow to the affected intestinal segment, resulting in reduced bowel perfusion and necrosis of the intestinal wall [11]. This progression substantially increases the risk of septic peritonitis, systemic inflammation, and death [11]. Recognizing these complications early is essential to prevent fatal outcomes.

While abdominal radiographs and ultrasounds (US) are frequently used as initial diagnostic modalities, both have prominent limitations. Radiographic findings may remain inconclusive because many small intestinal diseases fail to produce detectable changes, particularly in cases of partial obstruction [12]. US, while effective in detecting signs of bowel dilation, is limited in its ability to evaluate the entire trajectory of dilated bowel loops [13]. Additionally, the quality of acquired images can be influenced by patient-specific factors, including body size and the quantity of intra-peritoneal fat [1]. Furthermore, the accuracy of a US is highly dependent on operator expertise and can be significantly hindered by intestinal gas [13,14]. In cases of partial obstruction caused by foreign bodies, including bezoars, incomplete obstruction often leads only to increased intestinal motility from irritation, without presenting other typical signs of obstruction [12]. In contrast, CT scans provide significant advantages. They offer high diagnostic accuracy and valuable surgical guidance owing to their detailed anatomical visualization and multiplanar capabilities [10,15]. CT has recently been employed to diagnose small intestinal obstructions in dogs and cats, demonstrating its accuracy in distinguishing obstructive from nonobstructive intestinal content [12]. This is largely owing to its ability to eliminate organ superimposition through reformatted imaging, which provides a comprehensive view of the abdomen [12]. Moreover, CT precisely identifies the location and level of bowel lesions and, in most cases, determines the underlying cause of obstruction [10]. To the best of our knowledge, comprehensive research that categorizes CT features in different types of gastrointestinal foreign bodies and examines associated complications using CT imaging remains limited in veterinary medicine. This knowledge gap is particularly critical given the potential for serious complications and the diagnostic challenges that these conditions present.

Therefore, this study aims to describe and compare the qualitative and quantitative CT features of various gastrointestinal foreign bodies, including bezoars and distinct foreign bodies, and identify CT-detectable complications associated with these foreign bodies as well as the factors influencing their occurrence to determine which complications or imaging features are related to adverse outcomes.

## 2. Materials and Methods

### 2.1. Study Design and Case Selection

This was a retrospective, multicenter study. Medical records from four animal hospitals (Konkuk University Veterinary Medical Teaching Hospital (Seoul, Republic of Korea), Daegu Animal Medical Center (Daegu, Republic of Korea), SD Animal Medical Center (Seoul, Republic of Korea), and Haeden Animal Medical Center (Yangju-si, Republic of Korea)) were reviewed. Medical records were searched from January 2016 to June 2024. Medical records were searched using terms related to the gastrointestinal foreign bodies and clinical signs, such as vomiting, diarrhea, or anorexia. The inclusion criteria were as follows: (1) patients who presented with clinical signs of gastrointestinal distress (e.g., decreased appetite, vomiting, or diarrhea) and gastrointestinal foreign bodies considered as a differential diagnosis; (2) those who underwent a CT examination, including at least a pre-contrast abdominal CT; and (3) those with foreign bodies confirmed using endoscopy or surgical interventions. Additionally, only cases with both radiographic and US examinations available were included in the analysis of imaging characteristics. Cases in which foreign bodies were incidentally found on CT or were determined to have no clinical correlation owing to the absence of obstruction were excluded. As this was a retrospective study, no prior sample size calculation was performed. All eligible cases meeting the inclusion criteria were reviewed. Ethical approval was not required, as the study was retrospective in nature and did not involve any experimental interventions or manipulation of animals. Owner consent for the use of clinical and imaging data was obtained at the time of hospital admission.

### 2.2. Imaging Examinations and Modalities

CT examinations were performed using the Brilliance (General Electric Medical systems) (Philips Healthcare, Amsterdam, The Netherlands), Toshiba Aquilion CX 128-slice CT scanner (Canon Medical Systems Corporation, Otawara-shi, Japan), and Aquilion Lightning 160 (Canon Medical Systems Corporation, Otawara-shi, Japan). The number of detector rows in the CT scanners ranged from 64 to 160. The acquisition parameters for the CT images included a slice thickness of 1–1.5 mm, helical pitch of 0.8–1.375, rotation time of 0.6–1 s, kVp value of 120, mAs value ranging from 70 to 200, and variable field of view and matrix dimensions. The dogs and cats were positioned in either sternal or dorsal recumbency. A contrast study was conducted using a nonionic contrast medium (iohexol, 350 mg iodine/mL; Omnipaque, GE Healthcare, Chicago, IL, USA) administered either manually or with a power injector at a rate of 2–2.2 mL/s through the cephalic vein. The dose range of contrast medium was 2.5–3 mL/kg. Postcontrast images were acquired 60–80 s after contrast injection.

Abdominal radiographs were obtained using a digital X-ray system (IRE-HF500, iRE Tech, Phnom Penh, Cambodia) with typical acquisition parameters of 55–65 kVp and 2–5 mAs, adjusted according to patient size and body condition. US examinations were performed using a Philips ultrasound system with a high-resolution linear probe (Hitachi, Tokyo, Japan, L12-5; frequency range 5–12 MHz). Acoustic output was kept below 100%, and imaging parameters were optimized for each patient. Key radiographic and US findings were analyzed and interpreted by a veterinary radiologist (J.K.) under the supervision of a senior radiologist with 30 years of experience in veterinary diagnostic imaging.

### 2.3. Data Collection and Imaging Evaluation

Patient data—including age, breed, sex, body weight, and the owner’s primary concerns regarding gastrointestinal foreign bodies—were obtained from medical records. The duration of clinical signs was determined based on either the known ingestion event or the onset of clinical symptoms when ingestion was not witnessed [7]. All imaging data, including radiographs, US images, and CT scans, were reviewed using commercially available software (RadiAnt DICOM Viewer, Medixant, Poznań, Poland; available online: https://www.radiantviewer.com/, accessed on 10 January 2025).

Radiographic evaluation included assessment of small intestinal diameter and the presence of foreign bodies. Dilation was considered significant when the ratio of small intestinal diameter to the height of the L5 vertebral body in dogs exceeded 1.6 or the L2 endplate in cats exceeded 2.0 [16,17].

US evaluation focused on detecting acoustic shadowing, surface textures, and dilation of bowel segments, which were compared with CT findings to confirm obstruction and foreign body presence.

**Figure 1:**
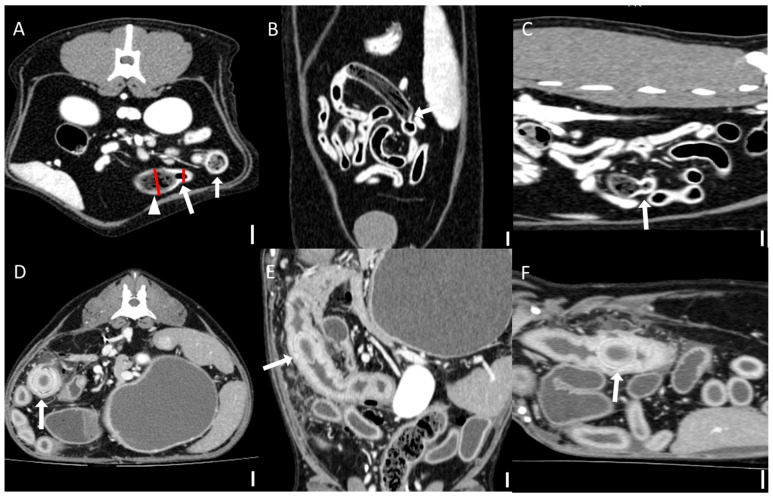
Representative computed tomographic images of the transition zone and measurement of small intestinal diameter across transition zone (scale bar equals 1 cm). The presence of a transition zone and measurements of small intestinal diameter were evaluated by multiplanar reconstruction. (**A**–**C**) Multiplanar CT images ((**A**): transverse, (**B**): dorsal, (**C**): sagittal) from a case of jejunal obstruction caused by trichobezoars in the jejunum (arrowhead). An abrupt change in intestinal diameter (arrow) can be seen, indicative of a transition zone. The small intestinal diameter was measured at the most distended segment proximal (orad) to the foreign body and at the adjacent collapsed distal (aborad) segment. Red lines indicate the measurement sites used to calculate the diameter ratio. (**D**–**F**). CT images ((**D**): transverse, (**E**): dorsal, (**F**): sagittal) from a case of a duodenal foreign body, identified as a fruit pit (arrow), without a visible transition zone.

**Figure 2:**
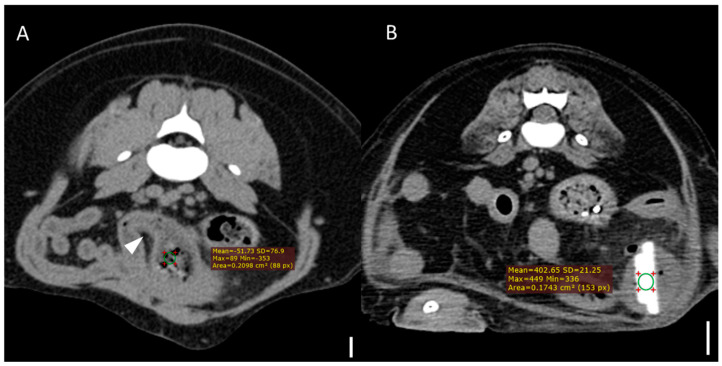
Representative CT images demonstrating attenuation measurement using region-of-interest (ROI) analysis (scale bar equals 1 cm) (**A**) Transverse CT image from a case of bezoar obstruction. The arrowhead indicates the presumed site of obstruction in the jejunum. A circular ROI (area = 20.98 mm^2^) was manually placed over the obstructive material, yielding a mean attenuation value of –51.73 Hounsfield units (HUs). (**B**) Transverse CT image from a case with a distinct foreign body (stone), where the ROI (area = 17.43 mm^2^) was placed directly on the foreign body, yielding a mean attenuation of 402.65 HUs. All measurements were performed on precontrast images using manually drawn circular ROIs, with consistent size (15–20 mm^2^) across cases.

**Figure 3:**
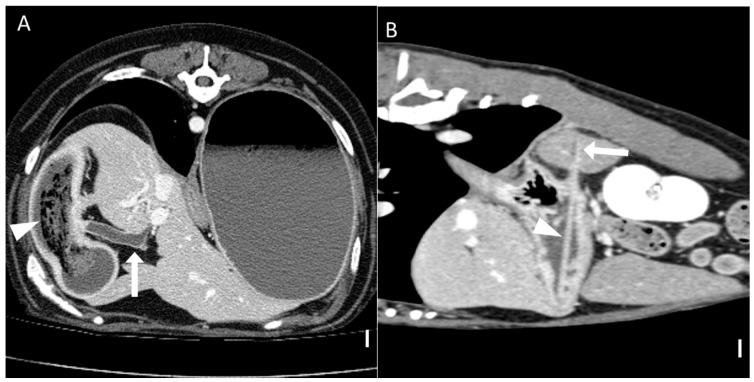
Representative CT images of foreign bodies affecting organs beyond the gastrointestinal tract (scale bar equals 1 cm) (**A**) Transverse CT image showing a dilated (8.6 mm) common bile duct (arrow) due to obstruction of the major duodenal papilla caused by a foreign body. The patient has duodenal obstruction resulting from a trichobezoar (arrowhead). (**B**) Sagittal CT image demonstrating a foreign body (arrow) penetrating the stomach wall and extending into the spleen (arrowhead). The patient underwent gastrotomy and splenectomy, and the foreign body was identified as a toothpick. CT, computed tomography.

Computed tomography (CT) images were evaluated using multiplanar reconstruction tools provided by Radiant software. In most cases, postcontrast images were available; however, precontrast images were used specifically for attenuation value (HU) measurements of foreign bodies. CT evaluations included both qualitative and quantitative assessments. Qualitative CT criteria included the presence of a transition zone, defined as an area where the bowel abruptly changes from dilated to normal; collapsed segments [18] (Figure 1); the identification of the boundary between foreign bodies and intestinal contents; and the location of the foreign bodies. Gastric foreign bodies were excluded from transition zone evaluation, as the stomach does not exhibit a discrete diameter change consistent with the transition zone definition, which applies specifically to small bowel obstruction [19]. The presence of a boundary between the foreign body and the small intestinal contents was also evaluated through multiplanar reconstruction. The location of foreign bodies was categorized as follows: stomach, duodenum (the portion of the intestine extending aborally from the stomach to the proximal jejunum situated to the left of the mesenteric root), jejunum (the section of intestine between the aborad broad portion of the duodenum and ileum), ileum (the distal portion of the small intestine located abroad to ileocolic junction), and multisegmental involvement [4]. The quantitative CT evaluation criteria included the mean, maximum, and minimum attenuation values of the foreign bodies; the ratio of the small intestinal diameter between the most distended segment proximal (orad) to the foreign body and the adjacent collapsed distal (aborad) segment [15], (Figure 1); the number of complications identified on CT imaging caused by foreign bodies; and the severity of small intestinal dilation caused by foreign bodies normalized to vertebral measurements (SI/L5 in dogs and SI/L2 endplate in cats). Attenuation value measurements were conducted on precontrast images, with values measured in Hounsfield units (HUs) within manually drawn circular regions of interest typically 15–20 mm^2^ in area (Figure 2). For distinct foreign bodies, ROIs were placed directly within the visible object. In cases of bezoars, measurements were taken from the area presumed to contain the foreign material, based on the site of obstruction identified on CT. ROIs were carefully positioned to avoid intraluminal gas or the adjacent intestinal wall, and three measurements were averaged for each case. Complications identified on CT imaging included signs of peritonitis (e.g., increased mesenteric density and peritoneal fluid) [20], rupture of the bowel wall, involvement of foreign bodies across multiple segments [21], foreign bodies affecting organs beyond the gastrointestinal tract (Figure 3), signs of bowel wall ischemia or hypoxic changes, and the degree of obstruction (complete or partial). The severity of small intestinal dilation was classified as normal (SI/L5 < 1.6 in dogs, SI/L2 < 2.0 in cats), mild (SI/L5 1.6–2.0 in dogs, SI/L2 2.0–2.5 in cats), moderate (SI/L5 2.0–2.4 in dogs, SI/L2 2.5–3.0 in cats), or severe (SI/L5 > 2.4 in dogs, SI/L2 > 3.0 in cats) [5,16,17,22]. This measurement was applied exclusively to small intestinal segments; gastric foreign bodies were excluded from this assessment.

Foreign body classification was based on surgical or endoscopic findings. Objects with clear and discrete outlines matching the CT appearance were classified as distinct foreign bodies. Materials lacking well-defined contours, often mixed with luminal contents, were classified as bezoars. Bezoars were further categorized into trichobezoars (composed of hair or fibers) or foreign body bezoars (composition not clearly identified).

An adverse group was defined as either death of the patient or the need for reoperation due to severe peritonitis or unresolved clinical symptoms following the initial surgery.

### 2.4. Statistical Analysis

Statistical analyses were performed using SPSS 27.0 (IBM SPSS Statistics, New York, NY, USA). The normality of all continuous variables—including age, body weight, and attenuation values (HUs)—was assessed using the Shapiro–Wilk test prior to statistical comparison. Normally distributed data were analyzed using the independent samples T-test, while non-normally distributed data were analyzed with the Mann–Whitney U test or Kruskal–Wallis test. The Mann–Whitney U test was applied to compare attenuation values (HUs), the ratio of small intestinal diameters between proximal and distal dilated segments, and symptom duration. The Kruskal–Wallis test was used to compare the number of complications across foreign body locations. Categorical variables, including foreign body location and the presence of a transition zone, were analyzed using Fisher’s exact test, while the boundary of foreign bodies was evaluated using Pearson’s Chi-square test. A *p*-value of <0.05 was considered statistically significant.

## 3. Results

### 3.1. Study Population

A total of 49 cases (35 dogs and 14 cats) were initially reviewed, of which 33 (26 dogs and 7 cats) met the inclusion criteria. CT examinations were performed using either pre-contrast (*n* = 4) or postcontrast (*n* = 29) protocols. Among these 33 cases, foreign bodies retrieved through endoscopy or surgery were classified into 15 bezoars and 18 distinct foreign objects. Of the 15 patients with bezoars, 6 underwent all three imaging modalities: CT, radiography, and US.

The dog population consisted of Maltese (*n* = 6), Poodles (*n* = 4), Golden Retrievers (*n* = 2), Samoyeds (*n* = 2), French Bulldogs (*n* = 2), and others (*n* = 10; one each of King Charles Spaniel, Doberman, Dachshund, Boston Terrier, Shih Tzu, German Shepherd, Papillon, Labrador Retriever, Border Collie, and Miniature Pinscher). The cat breeds included the Norwegian Forest cat (*n* = 2) and others (*n* = 5; one each of Russian Blue, Korean Shorthair, Turkish Angora, Bengal cat, and mixed breed).

The mean age at presentation was 9.06 ± 3.94 years (range, 1–17 years). The study population included 19 male animals (15 castrated, 4 intact) and 14 female animals (11 spayed, 3 intact).

The most common clinical signs were vomiting and anorexia, observed in 10 animals, while less common signs included diarrhea (*n* = 2), hematochezia (*n* = 1), inguinal edema (*n* = 1), and cutaneous abscess formation (*n* = 1).

The duration of clinical signs differed significantly between the bezoar and distinct foreign body groups (*p* = 0.013). The median duration was 14 days (interquartile range [IQR], 11–25 days) for the bezoar group and 5.5 days (IQR, 6.5–13 days) for the distinct foreign body group. Additionally, the sex distribution significantly differed between the two groups (*p* = 0.002). However, there were no significant differences in age, body weight, or specific clinical signs between the groups (*p* > 0.05).

### 3.2. CT Characteristics of Foreign Bodies

The foreign bodies retrieved using endoscopy or surgery included 15 bezoars and 18 distinct foreign objects (Table 1). The following 18 with distinct foreign objects were identified as six plastic materials, four fruit pits, four pointed wooden sticks (two toothpicks, two wooden skewers), two metal objects (one bottle cap, one needle), one stone, and one bone fragment. Of the 15 patients with bezoars, 10 were trichobezoars, and 5 were foreign body bezoars (2 plastic bags, 1 memory foam, 1 silicone, and 1 composed of a mixture of metal and phytobezoar).

**Figure 4:**
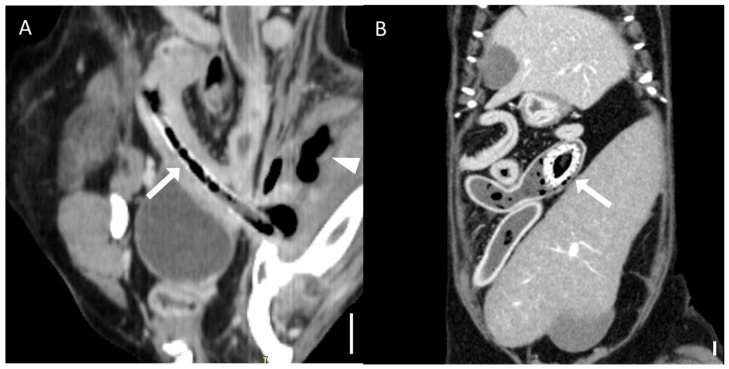
Representative CT images of foreign bodies with tubular-like appearance (scale bar equals 1 cm). (**A**) Dorsal CT image showing a foreign body (arrow) with a tubular-like shape in the jejunum, causing obstruction and perforation, accompanied by subcutaneous emphysema (arrowhead). The foreign body is a straw-shaped silicone object. (**B**) Dorsal CT image demonstrating a tubular-like foreign body (arrow), identified as a plum pit, causing complete jejunal obstruction. CT, computed tomography.

All distinct foreign bodies were visible on CT imaging. Among the six plastic foreign bodies, two (16.6%) exhibited heterogeneous attenuation and were classified as having a tubular-like appearance (Figure 4). This appearance is characterized by a hypoattenuating center surrounded by a well-defined hyperattenuating rim [23]. One was a plastic ball with a hollow space, and the other one was a straw-like plastic structure. The mean attenuation values were variable. All four cases of fruit pits demonstrated heterogeneous attenuation and exhibited a tubular-like appearance. Four pointed wooden sticks showed homogeneous attenuation, with one case exhibiting fat attenuation and the remaining cases demonstrating hyperattenuation relative to soft tissue.

**Figure 5:**
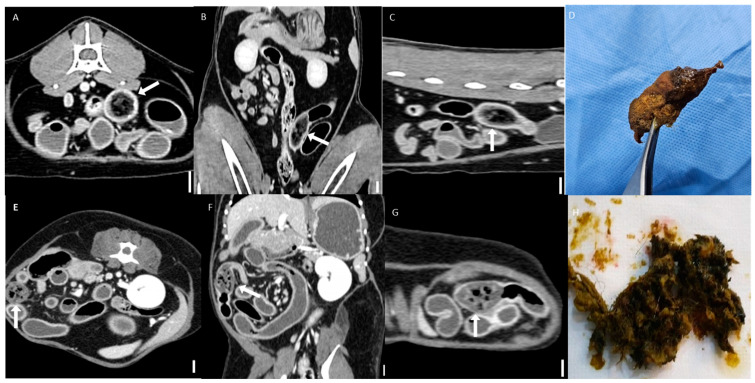
Representative CT images of bezoars (scale bar equals 1 cm). (**A**–**C**) Multiplanar CT images of a jejunal bezoar: (**A**) transverse, (**B**) dorsal, and (**C**) sagittal views. The bezoar (arrows) appears as an intraluminal mass with a mottled gas pattern. (**D**) The corresponding surgically removed bezoar was identified as a trichobezoar composed of hair. (**E**–**G**) Multiplanar CT images of another jejunal bezoar: (**E**) transverse, (**F**) dorsal, and (**G**) sagittal views. The bezoar (arrows) exhibits similar mottled gas appearance. (**H**) The corresponding foreign body was identified as a trichobezoar primarily composed of synthetic fibers, likely originating from toy fragments.

All bezoars were mostly classified into the suspected group based on CT imaging and displayed heterogeneous attenuation. They typically appeared as intraluminal masses with a mottled gas pattern, characteristic of their heterogeneous internal structure (Figure 5). Two cases—one in the trichobezoar group and one in the foreign body bezoar group—were visible on CT imaging and exhibited distinct boundaries. A comprehensive analysis of specific HU values is presented in Section 3.5.

### 3.3. Radiographic and Ultrasonographic Characteristics of Bezoar

**Figure 6:**
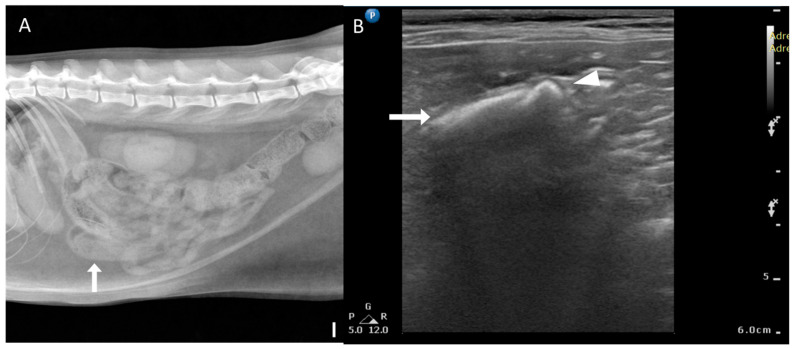
Radiographic and ultrasonographic features of bezoars. (**A**) Right lateral radiograph (scale bar equals 1 cm) of a patient with a trichobezoar. Focal small intestinal dilation and a mottled solid appearance are observed in the small intestine (arrow). (**B**) Ultrasonographic image of the same patient as in (**A**), showing a heterogeneous surface with mixed hypoechoic and hyperechoic parenchyma (arrow) in the impacted segment. A trichobezoar in the jejunum is seen with acoustic shadowing (arrowhead).

Radiographic and US findings of bezoars are summarized in Table 2. Foreign bodies (1/6, 16.7%) with a mottled and solid appearance (Figure 6) were identified in only one case using radiography. Nonspecific signs of mechanical obstruction, such as small intestinal dilation, were observed in most cases (5/6, 83.3%). On US, acoustic shadowing was detected in most cases (4/6, 66.7%), and a heterogeneous surface (Figure 6) was noted in areas suspected of containing foreign bodies. In two cases, acoustic shadowing was absent. All cases exhibited dilation of small bowel segments, which is a nonspecific indicator of small bowel obstruction.

### 3.4. Qualitative CT Features

**Figure 7:**
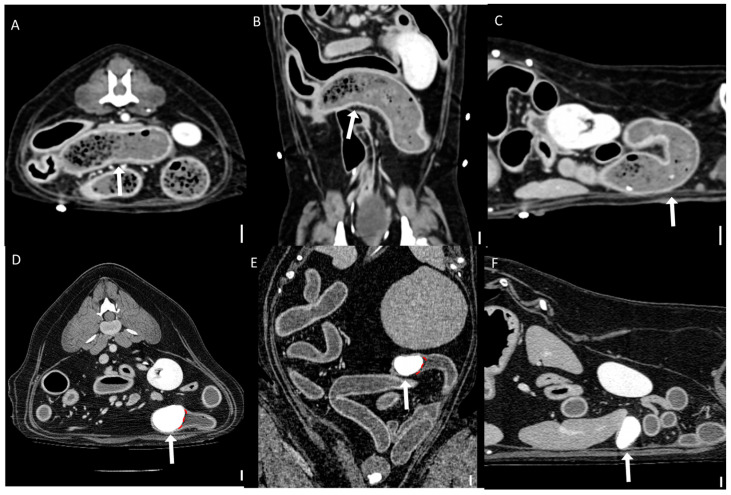
Representative CT images of the boundary between foreign bodies and intestinal contents (scale bar equals 1 cm). (**A**–**C**) Multiplanar CT images showing an unclear boundary between a foreign body and the surrounding intestinal contents: (**A**) transverse, (**B**) dorsal, and (**C**) sagittal views. The obstruction was caused by a bezoar (arrows) identified as memory foam. (**D**–**F**) Multiplanar CT images showing a clear boundary (dashed line) between the foreign body and the surrounding intestinal contents: (**D**) transverse, (**E**) dorsal, and (**F**) sagittal views. The obstruction was caused by a stone (arrows).

The qualitative CT findings are summarized in Table 3 and Table 4. A transition zone was observed in 92.3% (12/13) of bezoar cases and 41.7% (5/12) of distinct foreign body cases (*p* = 0.011). Cases with gastric foreign bodies were excluded from this analysis due to the ambiguity of transition zone identification in the stomach. Clear boundaries between the foreign body and intestinal contents were significantly more common in the distinct foreign body group (94.4%, 17/18) than in the bezoar group (33.3%, 5/15) (*p* < 0.001; Figure 7). There was no significant difference in the anatomical location of foreign bodies between the groups (*p* = 0.625), with the jejunum being the most frequently affected segment in both (46.7% of bezoars and 38.9% of distinct foreign bodies).

### 3.5. Quantitative CT Features

The quantitative CT findings are summarized in Table 5. Compared to distinct foreign bodies, bezoars exhibited significantly lower attenuation values across all three measures. Median values in the bezoar group were –61.2 HUs (average), 53 HUs (maximum), and –364 HUs (minimum), while the distinct foreign body group showed 166.8 HUs, 254.5 HUs, and 139.5 HUs, respectively (*p* < 0.001, *p* < 0.001, and *p* = 0.004).

The median ratio of the small intestinal diameter (proximal (orad) to distal (aborad) across transition zone) was significantly higher in the bezoar group (2.9 [IQR, 2.1–3.7]) than in the distinct foreign body group (1.25 [IQR, 0.2–2.31]) (*p* = 0.012). Cases with gastric foreign bodies were excluded from this analysis due to the subjective nature of evaluating gastric dilation.

No significant differences were observed between the two groups in the number of CT-identified complications or in the severity of intestinal dilation normalized to vertebral length.

### 3.6. Complications and Adverse Outcome Group

The number of complications identified on CT compared to the location of foreign bodies is summarized in Table 6. The median numbers of complications for foreign bodies located in the stomach, duodenum, jejunum, and multiple segments were 1.0 (IQR, 0.5–2.0), 4.0 (IQR, 3.75–4.0), 2.0 (IQR, 1.5–2.5), and 2.5 (IQR, 1.12–2.87), respectively. There was a significant difference among these groups (*p* = 0.015).

Among the 33 dogs and cats included in the study, 6 cases (18%) were categorized into the adverse outcome group, comprising 5 deaths and 1 reoperation (non-fatal case). To identify factors associated with adverse outcomes, Fisher’s test was used to compare cases in the adverse outcome group with those without adverse outcomes. Bowel wall rupture was significantly associated with the adverse outcomes group (*p* < 0.001), while no other complications showed a significant association. Furthermore, the location of the foreign body was not significantly associated with adverse outcomes (*p* > 0.05).

## 4. Discussion

To the authors’ knowledge, this study is the first to comprehensively characterize the CT features of bezoars in comparison with other types of gastrointestinal foreign bodies in dogs and cats. Bezoars exhibit less distinct boundaries on CT compared to distinct foreign bodies, making them more challenging to diagnose, particularly because they often resemble fecal material within the colon [24].

The presence of a transition zone and a clear difference in diameters between the proximal and distal bowel segments across the transition zone were identified as indicators of gastrointestinal obstruction caused by bezoars. The significantly higher proximal-to-distal bowel diameter ratio observed in bezoar cases may be partly explained by the prolonged duration of clinical signs in these patients, which was statistically significant compared to those with distinct foreign bodies. Extended obstruction likely led to progressive bowel dilation proximal to the bezoar, intensifying the contrast between the dilated and collapsed segments. Furthermore, the distinct composition of bezoars, typically consisting of fibers or hair, may contribute to their tendency to adhere to or mold against the surrounding intestinal wall, potentially resulting in a more stable and complete obstruction [8]. This may also explain why transition zones are more consistently observed in bezoar-induced obstructions. Additionally, the diagnostic challenge posed by bezoars, particularly their subtle or atypical appearance on radiographs and US, may have delayed definitive diagnosis and treatment, thereby exacerbating the obstruction over time. This phenomenon has also been documented in human medicine [15,18] and often necessitates surgical intervention in both veterinary and human cases. On CT, bezoars often resemble surrounding intestinal contents due to their fat attenuation values, and they may be mistaken for the small bowel feces sign, which typically spans a longer segment and is not located in the transition zone [24,25,26]. This diagnostic ambiguity highlights the importance of identifying a transition zone and a marked difference in bowel diameters when suspecting bezoar-induced obstruction, as has been similarly reported in the literature on humans [24,27].

Foreign bodies located in the duodenum were associated with a higher incidence of complications in this study, and bowel wall rupture was significantly linked to fatal outcomes. This can be explained by the complex anatomy of the duodenal region, which is closely related to the pancreas and bile ducts [28,29]. Duodenal obstruction may lead to severe secondary conditions, such as extrahepatic biliary obstruction and pancreatitis. Surgical management of duodenal lesions is also more complex and may require procedures such as cholecystoenterostomy, gastrojejunostomy, or pylorectomy in cases of extensive damage, often resulting in a poor prognosis [30]. Furthermore, bowel wall rupture, a complication significantly associated with adverse outcomes in this study, can lead to peritonitis, systemic inflammation, and septic shock [21,31,32].

The inclusion of various breeds and a wide age range enhances the clinical relevance and applicability of the findings to diverse veterinary patients. Although a difference in sex distribution was identified between groups, this may reflect sampling bias rather than a true clinical association with foreign body ingestion.

Several studies have discussed the accuracy of different imaging modalities in diagnosing foreign body obstruction in both human and veterinary medicine [10,13]. As discussed earlier, abdominal radiography and US have significant limitations. For instance, one study reported that 30% of mechanical obstruction cases went undetected on radiographs [33]. Additionally, unmineralized or nonmetallic foreign objects are much more difficult to identify on X-rays [12]. Regarding US, although signs of small bowel obstruction can often be visualized, the exact location or cause of the obstruction might remain undetected [13]. As highlighted in this study, identifying the transition zone is an important feature when diagnosing foreign body-induced obstruction, and the inability to reliably detect this zone using US could pose diagnostic challenges. Furthermore, patient-related factors can complicate the diagnosis. For example, in large-breed dogs weighing over 25 kg, CT is more advantageous than US in screening for abdominal diseases [34]. Factors such as body size and the amount of intraperitoneal fat can significantly affect the quality of US images [1].

CT is widely regarded as the imaging modality of choice for diagnosing gastrointestinal obstruction, particularly in pediatric patients in human medicine [35]. Similarly, in veterinary medicine, CT has been shown to outperform radiography and US in both sensitivity and specificity for detecting mechanical obstruction [34,36,37,38]. One study reported that CT achieved 100% sensitivity and specificity, whereas US, although highly sensitive, had a lower specificity of 67% [34]. These findings highlight CT’s diagnostic precision and its role in preventing unnecessary surgical interventions. Consistent with previous studies, this study supports the use of CT with multiplanar reconstruction, as its ability to explore the entire trajectory of dilated bowel loops can greatly aid in identifying the obstruction site.

In the case of bezoars, although limited studies are available in veterinary medicine, extensive research has been conducted in human medicine. Bezoars exhibit characteristic imaging features, appearing as mottled radiolucent material with solid matter on radiographs and as hyperechoic arc-like structures with acoustic shadowing on US [24]. However, in the same study, only 18% of radiographs demonstrated the typical appearance of bezoars, and US findings were confirmed during surgery in only 58% of cases. Similarly, in this study, radiographic detection was rare, and most US cases displayed heterogeneous surfaces rather than clear acoustic shadowing. This discrepancy may be attributed to species-specific differences, imaging protocols, or variations in bezoar composition. These findings offer valuable insights into the radiographic and US characteristics of bezoars, contributing to the limited body of veterinary literature.

The results offer new insights into the challenges of diagnosing bezoars on CT and emphasize their clinical significance in veterinary medicine. By systematically categorizing and analyzing gastrointestinal foreign body types and their associated complications, this study fills an important gap in the veterinary literature and highlights the necessity of careful CT evaluation to improve diagnostic precision. Key diagnostic indicators of bezoars include the presence of a transition zone and differences in the diameters of proximal and distal bowel segments, which are particularly useful when foreign bodies are difficult to identify. Furthermore, early detection of duodenal foreign bodies is essential due to their higher risk of severe complications, and bowel wall rupture requires immediate surgical intervention because of its strong association with adverse outcomes. By employing CT imaging, clinicians can better assess the severity of obstructions, recognize potential complications, and determine appropriate treatment strategies, ultimately improving clinical outcomes in affected animals.

Although the sample size of this study was limited (*n* = 33), the data exclusively included cases confirmed to have signs of obstruction through endoscopic or surgical intervention. Furthermore, the cases were represented by the small, medium, and large dog breeds (*n* = 15, *n* = 5, *n* = 6, respectively) as well as cats (*n* = 7), which enhanced the representativeness of the findings and supported their applicability to small animal veterinary practice. The diversity in the sample reflects the heterogeneity seen in clinical practice and strengthens the generalizability of the results.

This study has some limitations. First, the study exclusively included cases with confirmed mechanical obstructions; consequently, functional ileus cases, although clinically relevant, were not considered. This limits the generalizability of our findings to all causes of gastrointestinal obstruction. Second, while the study analyzed the number of complications, it did not assess their severity or long-term clinical impact. Future studies should aim to establish a grading system to evaluate complication severity. Third, the inclusion of both dogs and cats in the analysis may have introduced species-specific variations that were not fully accounted for. Differences in gastrointestinal anatomy and physiological responses between species could influence obstruction severity and diagnostic accuracy. Finally, the limited number of radiographs and US exams of bezoars restricted our ability to perform statistical analyses on these modalities. Moreover, contrast-enhanced radiographs were not included, as they were inconsistently available in the retrospective dataset and generally omitted in cases scheduled for CT. Future studies should include larger datasets to determine the true diagnostic value of radiography and US for bezoar detection. Further research should also compare bezoars with other gastrointestinal conditions, such as the small- bowel feces sign, and explore species-specific variations to develop more tailored diagnostic and treatment strategies for dogs and cats.

## 5. Conclusions

This study highlights the superior diagnostic utility of CT in identifying gastrointestinal foreign bodies, particularly bezoars, which are often challenging to detect using radiography or US. Key CT features, such as the presence of a transition zone and the ratio of intestinal diameters across the transition zone, provided valuable indicators for obstruction and severity. Early CT evaluation is especially important in cases involving duodenal foreign bodies or bowel wall rupture, as these were associated with a higher risk of complications and mortality. These findings underscore the role of CT in guiding timely clinical decision making and improving outcomes in veterinary emergency care.

## Figures and Tables

**Table 1:** CT features of foreign bodies in 33 dogs and cats, by foreign body type.

Type of Foreign Body	Total (*n* = 33)	Identified/Suspected on CT	Homogeneity	Mean Attenuation
Plastic	6	Identified 6 Suspected 0	Homogenous 4 Heterogenous 2	Bone 3 Hyperattenuating to soft tissue 1 Tubular-like 2
Fruit pit	4	Identified 4 Suspected 0	Homogenous 0 Heterogenous 4	Tubular-like 4
Pointed wooden pick	4	Identified 4 Suspected 0	Homogenous 4 Heterogenous 0	Hyperattenuating to soft tissue 3 Fat 1
Metal	2	Identified 2 Suspected 0	Homogenous 2 Heterogenous 0	Bone 1 Hyperattenuating to soft tissue 1
Stone	1	Identified 1 Suspected 0	Homogenous 1 Heterogenous 0	Bone 1
Bone fragment	1	Identified 1 Suspected 0	Homogenous 1 Heterogenous 0	Bone 1
Trichobezoar	10	Identified 2 Suspected 8	Homogenous 0 Heterogenous 10	Fat 8 Fluid 2
Foreign body bezoar	5	Identified 1 Suspected 4	Homogenous 0 Heterogenous 5	Fat 4 Hyperattenuating to soft tissue 1

Mean attenuation. Air: −1000 HUs, fat: −50 to −200 HUs, fluid: 0 to 20 HUs, soft tissue: 20 to 60 HUs, hyperattenuating to soft tissue: 60 to 400 HUs, bone: 400 HUs and above, tubular-like appearance: hyperattenuating rim, hypoattenuating center. CT, computed tomography; HUs, Hounsfield units.

**Table 2:** Radiographic and ultrasonographic features of bezoars (*n* = 6).

	Radiographic Features	Ultrasonographic Features
1	Focal small intestinal dilation	Arc-like surface with acoustic shadowing
2	Focal small intestinal dilation	Heterogenous surface with acoustic shadowing
3	No significant abnormalities	Heterogenous surface with acoustic shadowing
4	General small intestinal dilation	Heterogenous surface without acoustic shadowing
5	Mottled, solid foreign bodies in small intestine; focal small intestinal dilation	Heterogenous and arc-like surface with acoustic shadowing
6	Focal small intestinal dilation	Heterogenous surface without acoustic shadowing

**Table 3:** CT imaging variables comparing bezoars and distinct foreign bodies in terms of transition zone and presence of boundary.

	Bezoar (*n* = 13 *, 15)	Distinct Foreign Body (*n* = 12 *, 18)	*p*-Value
Transition zone	Present 12/13 (92.3%) Absent 1/13 (7.7%)	Present 5/12 (41.7%) Absent 7/12 (58.3%)	0.011
Boundary of foreign body	Present 5/15 (33.3%) Absent 10/15 (66.7%)	Present 17/18 (94.4%) Absent 1/18 (5.6%)	<0.001

All data are presented as numbers and percentages. * Cases where the transition zone is not visible, such as when the foreign body is in the stomach, are excluded from the sample. CT, computed tomography.

**Table 4:** Anatomical location differences between bezoars and distinct foreign bodies.

	Stomach	Duodenum	Jejunum	Multisegmental	*p*-Value
Bezoars	2/15 (13.3%)	2/15 (13.3%)	7/15 (46.7%)	4/15 (26.7%)	0.625
Distinct foreign body	6/18 (33.3%)	1/18 (5.6%)	7/18 (38.9%)	4/18 (22.2%)	

All data are presented as numbers and percentages.

**Table 5:** Comparison of mean, maximum, and minimum attenuation value between the bezoar and the distinct foreign body.

	Bezoar	Distinct Foreign Body	*p*-Value
Mean	−61.2 (−111.5 to −10.9)	166.8 (−40.8 to 374.4)	<0.001
Maximum	53 (−5 to 111)	254.5 (13.6 to 495.4)	<0.001
Minimum	−364 (−649 to −79)	139.5 (−191.2 to 470.2)	0.004

All data are presented as median with interquartile range.

**Table 6:** Comparison between the number of complications identified on CT and locations of foreign bodies.

	Stomach	Duodenum	Jejunum	Multisegmental	*p*-Value
Number	1.0 (0.5 to 2.0)	4.0 (3.75 to 4.0)	2.0 (1.5 to 2.5)	2.5 (1.12 to 2.87)	0.015

All data are presented as median with interquartile range CT, computed tomography.

## Data Availability

Data from this study are available upon reasonable request from the corresponding authors.
